# Comparing four diagnostic tests for *Giardia duodenalis* in dogs using latent class analysis

**DOI:** 10.1186/s13071-018-3014-2

**Published:** 2018-07-31

**Authors:** Mathilde Uiterwijk, Rolf Nijsse, Frans N. J. Kooyman, Jaap A. Wagenaar, Lapo Mughini-Gras, Gerrit Koop, Harm W. Ploeger

**Affiliations:** 1 Department of Infectious Diseases and Immunology, Faculty of Veterinary Medicine, Utrecht University, Utrecht, The Netherlands; 2 Present address: National Institute for Public Health and the Environment (RIVM), Center for Infectious Disease Control (CIb), Bilthoven, The Netherlands; 3 Wageningen Bioveterinary Research, Lelystad, The Netherlands; 4 National Institute for Public Health and the Environment (RIVM), Center for Infectious Disease Control (CIb), Bilthoven, The Netherlands; 5 Department of Farm Animal Health, Faculty of Veterinary Medicine, Utrecht University, Utrecht, The Netherlands

**Keywords:** Giardiasis, Canine, Prevalence, Diagnosis, Bayesian analysis

## Abstract

**Background:**

To accurately diagnose giardiosis in dogs, knowledge of diagnostic test characteristics and expected prevalence are required. The aim of this work was to estimate test characteristics (sensitivity and specificity) of four commonly used diagnostic tests for detection of *Giardia duodenalis* in dogs.

**Methods:**

Fecal samples from 573 dogs originating from four populations (household dogs, shelter dogs, hunting dogs and clinical dogs) were examined with centrifugation sedimentation flotation (CSF) coproscopical analysis, direct immunofluorescence assay (DFA, Merifluor *Cryptosporidium*/*Giardia*®), a rapid enzyme immunochromatographic assay (IDEXX SNAP *Giardia*®) and qPCR (SSU rDNA) for presence of *G. duodenalis*. Bayesian latent class analysis was used to determine test performance characteristics and to estimate *G. duodenalis* prevalence of each of the four dog populations.

**Results:**

All tests were highly specific. IDEXX SNAP *Giardia*® showed the highest specificity (99.6%) and qPCR the lowest (85.6%). The sensitivities were much more variable, with qPCR showing the highest (97.0%) and CSF the lowest (48.2%) sensitivity. DFA was more sensitive than IDEXX SNAP *Giardia*®, but slightly less specific. Prevalences of *G. duodenalis* differed substantially between populations, with the hunting dogs showing the highest *G. duodenalis* prevalence (64.9%) and the household dogs the lowest (7.9%).

**Conclusions:**

This study identifies qPCR as a valuable screening tool because of its high sensitivity, whereas methods using microscopy for cyst identification or cyst wall detection should be used in situations where high specificity is required. *G. duodenalis* is a prevalent gastro-intestinal parasite in Dutch dogs, especially in dogs living in groups (hunting and shelter dogs) and clinical dogs.

**Electronic supplementary material:**

The online version of this article (10.1186/s13071-018-3014-2) contains supplementary material, which is available to authorized users.

## Background

*Giardia* spp. are among the most prevalent parasites in dogs worldwide. The genus *Giardia* consists of six species, of which *Giardia duodenalis* (syn. *G. lamblia*, *G. intestinalis*) infects mammals such as humans, dogs, cats and cattle [1]. *Giardia duodenalis* has been further classified into eight assemblages (A to H), with various degrees of host specificity [1]. Dogs can be infected with *G. duodenalis* assemblages C and D and the potentially zoonotic assemblages A and B [2]. *Giardia* infection often is asymptomatic (giardiasis). If it becomes symptomatic (giardiosis), diarrhea is the most common symptom.

Several diagnostic tests for the detection of *G. duodenalis* are available. Detection of trophozoites and cysts with classic coproscopical methods and detection of coproantigens (cyst wall proteins) with rapid point of care immunochromatographic assays are the most commonly used tests in veterinary practice. Direct fluorescence assays have largely replaced classic coproscopical detection of cysts in veterinary research [3, 4]. More recently, molecular techniques have been developed, such as a quantitative real time PCR (qPCR) with a reportedly high sensitivity and specificity [5, 6].

Prevalences in dogs range between 0.4–34% in healthy adult dogs, 2.3–25% in dogs with signs of gastro-intestinal illness, 7–63% in puppies and 2.3–50% in dogs living in groups [7–17]. Several diagnostic tests with different test characteristics have been used to determine these prevalences [18]. To be able to compare these prevalences properly, insight is needed in the test characteristics.

Using a large cohort of dogs within four populations, the aim of this study was to compare test characteristics (sensitivity and specificity) of four commonly used tests for the detection of *G. duodenalis* in dogs. Additionally, prevalences for each dog population were estimated. The tests were a centrifugation sedimentation flotation (CSF) coproscopical technique, a direct immunofluorescence assay (DFA, Merifluor® *Cryptosporidium*/*Giardia*), a rapid enzyme immunochromatographic assay (IDEXX SNAP *Giardia*®) and a quantitative real time PCR (qPCR). Appointing one test as the gold standard will overestimate the characteristics of that test and as a consequence underestimate the sensitivity and specificity of the other tests. In order to obtain unbiased estimates of sensitivity and specificity, a Bayesian latent class analysis was used without assuming any test to be a perfect reference test.

## Methods

### Dogs

Fecal samples from in total 646 Dutch dogs belonging to four populations were collected between October 2013 and December 2014. Of these, 573 were tested with all four diagnostic tests. These 573 dogs are further subdivided below.

The household dogs consisted of 210 privately owned household dogs older than 6 months, participating in a study on *Toxocara canis* [19]. The dog owners collected the fecal samples and submitted them by mail.

The shelter dogs consisted of 137 shelter dogs from 16 shelter-kennels and the hunting dogs consisted of 34 dogs from two hunting-kennels. Fecal samples of the shelter and hunting dogs were collected by instructed personnel at the kennel or by veterinarians in training. The fecal samples were taken to the laboratory by the visiting veterinarians in training.

The clinical dogs consisted of 192 dogs for which a fecal sample was submitted to the Veterinary Microbiological Diagnostic Center (VMDC) of the Faculty of Veterinary Medicine of Utrecht University, The Netherlands, for endoparasite testing. Most fecal samples were sent in for diagnosing a possible parasitic cause of clinical symptoms and a few for control of therapy or for routine monitoring.

Fecal samples were processed as soon as possible after arrival, ranging from the same day to 5 days after collection. Because the fecal samples of the household and clinical dogs were sent in by mail, the interval between collection and processing of the samples was longer for the clinical and household dogs than for the shelter and hunting dogs.

### Centrifugation, sedimentation, flotation

Coproscopical analysis with CSF on fecal samples of household and group-housed dogs was performed as described previously [20]. In brief, 3–5 g of feces were suspended in approximately 55 ml of water and, after sieving, poured in a 12 ml centrifuge tube. Therefore, the contents of the tube corresponded to 0.6–1.0 g of feces. After centrifuging for 2 min at 1500× *g*, the supernatant was decanted. A sucrose solution (1.27–1.30 g/cm^3^) was added to the sediment, resuspended and centrifuged for 2 min at 1500× *g* with a coverslip on the meniscus. Then, the coverslip with approximately 50 μl of suspension attached to it, was removed upright and placed on a microscopic slide. For logistic reasons, approximately a third of the fecal samples of household and group-housed dogs were pooled. Here for, 3–5 g of each of the two samples were suspended in approximately 110 ml of water. When cysts were detected, the samples were both retested separately. The samples of the clinical dogs were examined in routine diagnostics setting by the VMDC, using ZnSO_4_ solution (1.34 g/cm^3^) instead of sucrose. The performance of both protocols was similar (data not shown). Microscopic slides were examined at 100× and 400× magnification. Cysts were semi-quantitatively scored with the use of a five class scoring system: -, none; +, a few in the whole slide; ++, several in the whole slide; +++, many; or ++++, very many in every field of view at 100× magnification.

The theoretical detection limit of the used CSF techniques were 1–1.7 cysts per gram feces.

### Direct immunofluorescence assay

One gram of feces was mixed well with 3 ml SAF (sodium acetate acetic acid formalin) and stored in the dark at room temperature until analysis. Cysts were detected with DFA, using the Merifluor® *Cryptosporidium*/*Giardia* kit (Meridian Bioscience Diagnostics Inc., Cincinnati, Ohio, USA) as described previously [3], with some minor modifications. In brief, the feces-SAF suspension was strained through a sieve to remove large debris. After centrifugation for 5 min at 1000× *g*, the supernatant was discarded and the sediment, if necessary, was filled up to 1 ml with distilled water and stirred. An aliquot of 10 μl was placed on a treated DFA slide with a 10 μl transfer loop. After staining, according to the manufacturer’s protocol, the slide was examined with a fluorescence microscope (Olympus, Tokyo, Japan; type BHS-F) for specific apple-green bright colored cysts, fitting size indication according to the manual of the test kit. The theoretical detection limit was 100 cysts per gram feces.

### Rapid enzyme immunochromatographic assay

For this assay, feces (0.5–1.0 g) were stored at -20 °C until analysis. The IDEXX SNAP *Giardia*® test (IDEXX Laboratories Inc., Westbrook, Maine, USA) was performed as described by the manufacturer after the feces aliquot was thawed to room temperature.

### Quantitative PCR

Feces (0.2 g) were stored at -20 °C until analysis. DNA was extracted with the QIAamp Fast Stool Mini Kit (Qiagen, Germantown, Maryland, USA) according to the manufacturer’s instructions with the following modifications. The extraction buffer was added to the frozen fecal sample after which the sample was thawed and suspended. The sample was subsequently frozen for 15 min at -80 °C. After thawing, the sample was heated for 5 min at 95 °C and the centrifugation step to obtain the supernatant was performed after proteinase K lysis. From the 100 μl eluted DNA, 5 μl was used for each PCR reaction. A 63 bp fragment of the SSU rDNA gene was amplified with qPCR [5]. Reactions were performed on Light Cycler 480 (Roche, Penzberg, Germany), with Phocine Herpes Virus as internal control. For the calibration curve and to determine cysts per gram feces (cpg), *Giardia* cysts from a CSF positive feces sample were purified by centrifugation on a sucrose cushion [21] and counted in a modified Fuchs-Rosenthal counting chamber. DNA was isolated from the cysts suspension as described above and was 10-fold serial diluted. The serial dilutions ranged from 3 × 10^6^ to 3 cysts per gram feces. Dilutions from 3 × 10^6^ to 300 cysts per gram feces always tested positive and yielded a linear relation between Cp and log-cyst concentration. A calibration curve was used from 3 × 10^6^ cysts per gram feces (= 2500 cysts per PCR reaction) with Cp values of 24.36–24.38 to 300 cysts per gram feces (0.25 cysts per PCR reaction) with Cp values of 38.96–39.43. Therefore, the cut-off Cp value was set on 40. Because a *Giardia* cyst is 16N and there are approximately 60 copies of rDNA in one genome, the theoretical detection limit of the qPCR was 1.25 cysts per gram feces.

To further investigate the performance of the qPCR, two additional experiments were conducted. First, all available samples positive with qPCR and negative with the other three diagnostic tests (*n* = 63) were amplified with the same primers, reagents and conditions as used for the qPCR, but run as a conventional PCR. Twenty randomly selected samples negative for all diagnostic tests, were run as negative control. Serial diluted purified cysts from two dog isolates were also run this way. Purification on sucrose cushion and counting was done as described above and after DNA isolation, the samples were 5-fold serial diluted. Secondly, to confirm that *G. duodenalis* was detected and not one of the other species of *Giardia*, all 35 samples from the shelter and hunting dogs which tested positive with the qPCR but negative with the other three tests, were subjected to the nested PCR on SSU rDNA [22]. The amplified products were sequenced and aligned to reference sequences from the GenBank database by Clustal W using the software Lasergene 12 (DNASTAR®, Madison, USA). The primers and probe of the qPCR [5] were also aligned this way.

### Statistical analyses

To estimate the test characteristics of the diagnostic tests (sensitivity and specificity) assuming that none of the tests can be seen as a gold standard, a latent class analysis was performed in a Bayesian context [23], simultaneously estimating the prevalence in each of the four dog populations. The parameters prevalence and characteristics of the tests were given uninformative priors. Conditional covariance was modeled between DFA and CSF, since they both rely on detecting cysts. Sensitivity and specificity for each test and their 95% PPI (posterior probability interval) were estimated. OpenBUGS, version 3.2.3 [24] was used for implementation of the model. The Markov chain Monte Carlo (MCMC) algorithm was run for 10,000 iterations and the first 1000 iterations were discarded as 'burn-in'. Convergence was assessed by running three MCMC chains in parallel and visually inspecting the time-series plots. Autocorrelation was assessed by inspecting the autocorrelation plots. The analysis was repeated whilst excluding the four dog populations, one at a time, to check for constant accuracy of tests across populations.

Differences in proportions of positive test results and the correlation between CPG and semi-quantitative detection of cyst shedding by CSF were assessed using the Chi-square, Fisher’s exact or two-sample Wilcoxon rank-sum test, as appropriate.

The level of agreement between the four tests was assessed using the kappa (ƙ) statistic. The ƙ outcome represents a slight (ƙ < 0.2), fair (0.2 ≤ ƙ ≤ 0.4), moderate (0.4 ≤ ƙ ≤ 0.6), substantial (0.6 ≤ ƙ ≤ 0.8) or almost perfect (ƙ > 0.8) agreement [25]. Statistical analyses were performed using STATA 13 (StataCorp LP, College Station, USA).

## Results

### Number of samples examined with each of the diagnostic tests

Of the 646 samples, 639 were tested with the CSF (59 positives), 645 were tested with the DFA (116 positives), 580 were tested with the IDEXX SNAP *Giardia*® test (86 positives), and all 646 were tested with the qPCR (189 positives). Overall, 573 samples were examined with all four tests. Data for all samples are provided in Additional file 1.

### Comparison of diagnostic tests

Table 1 shows the test results for the 573 samples that were tested with all four assays. In total, 420 samples of 573 (73.3%) had the same test result for all four diagnostic tests, either positive (*n* = 39; 6.8%) or negative (*n* = 381; 66.5%). There were no samples that tested negative with qPCR and positive with all three other tests.

**Table 1:** Cross-classified test results of four diagnostic tests for the detection of *G. duodenalis*

CSF	DFA	SNAP^a^	qPCR	No. of samples
+	+	+	+	39
+	+	+	-	0
+	+	-	+	9
+	-	+	+	2
-	+	+	+	30
-	-	+	+	9
-	-	-	+	70
-	+	-	+	13
+	-	-	-	1
+	+	-	-	0
+	-	+	-	0
+	-	-	+	5
-	-	+	-	2
-	+	-	-	10
-	+	+	-	2
-	-	-	-	381

^a^SNAP = IDEXX SNAP *Giardia*®

Table 2 shows the estimated sensitivities and specificities of the four tests according to the Bayesian latent class analysis. The qPCR showed the highest sensitivity and the CSF microscopy the lowest. DFA and IDEXX SNAP *Giardia*® were moderately sensitive. IDEXX SNAP *Giardia*®, however, was the most specific, closely followed by CSF microscopy and the qPCR had the lowest specificity. DFA was slightly less specific than IDEXX SNAP *Giardia*® and CSF microscopy. The overall ƙ between the four tests was 0.56 (95% CI: 0.52–0.61), which represents a moderate to substantial agreement.

**Table 2:** Estimated median sensitivities and specificities of the four tests with Bayesian latent class analysis

	Sensitivity (%)		Specificity (%)	
	Median	95% PPI^a^	Median	95% PPI
CSF	48.2	38.4–58.4	99.5	98.4–99.9
DFA	78.6	67.9–87.3	96.9	94.7–98.3
SNAP^b^	71.9	60.9–81.9	99.6	98.5–99.9
qPCR	97.0	92.1–99.5	85.6	81.7–89.3

^a^PPI = posterior probability interval

^b^SNAP = IDEXX SNAP *Giardia*®

The estimated prevalences of *G. duodenalis* in the four populations of dogs are presented in Table 3.

**Table 3:** Prevalences of *Giardia duodenalis* in four dog populations, estimated by Bayesian latent class analysis

Dog population	Median (%)	95% PPI^a^
Household	7.9	4.4–12.5
Shelter	21.0	14.4–29.3
Hunting	64.9	43.5–86.2
Clinical	24.7	18.8–31.3

^a^PPI = posterior probability interval

There was accordance between semi-quantitative detection of cysts with CSF and positivity of IDEXX SNAP *Giardia*®, DFA and especially qPCR. The higher the number of cysts detected with CSF, the more unlikely the other tests had a negative outcome. There was a significant correlation between CPG (determined with qPCR) and semi-quantitative cyst detection determined with CSF (*χ*^2^ = 49.3, *df* = 3, *P* = 0.0001). CSF tested positive at a higher median CPG (3.5 × 10^4^, IQR 1.1 × 10^3^–9.6 × 10^4^), compared to IDEXX SNAP *Giardia*® (2.5 × 10^4^, IQR 8.9 × 10^3^–6.9 × 10^4^) and DFA (1.9 × 10^4^, IQR 7.1 × 10^3^–5.8 × 10^4^).

### Specificity of qPCR

To gain more insight in the possible causes of the 70 uniquely qPCR positives (Table 1), we conducted two confirmation experiments on 63 of those samples (Additional file 2 and Additional file 3). Also, the primers and probe of the qPCR were aligned to GenBank *Giardia* sequences (Additional file 4) [26]. The uniquely qPCR positive samples run under conventional conditions (Additional file 2: Figure S1) resulted in amplification of the specific product in 86% of the samples, while all negative samples also were negative in the confirmation experiment [27]. Additional file 2: Figure S2 shows the gels of the serial diluted purified cysts from two dog isolates. The experiments and alignments showed that the qPCR is able to amplify DNA of *G. duodenalis* assemblages A to H and possibly *G. psittaci*. We found no indication that *G. duodenalis* assemblages E to H (false positive infection status) or *G. psittaci* (false positive test result) were detected with qPCR.

The median cpg of the 70 samples that were only positive with qPCR was 2.1 × 10^3^ (IQR 1.1 × 10^3^–6.5 × 10^3^), which was significantly lower (Wilcoxon rank-sum test, *Z* = 7.404, *P <* 0.0001) than the median CPG of all other qPCR positive samples (*n* = 119) being 1.8 × 10^4^ (IQR 6.6 × 10^3^–4.8 × 10^4^). Corresponding mean Cp values were 35.5 *versus* 32.1, respectively.

### *Giardia* cyst shedding based on qPCR results

There was a significant difference in the number of cysts shed (cpg) by dogs of different populations (*χ*^2^ = 21.8, *df* = 3, *P* = 0.0001). The qPCR positive clinical dogs (*n* = 64) showed the highest shedding of cysts (median cpg 2.5 × 10^4^, IQR 3.6 × 10^3^–8.5 × 10^4^) and the qPCR positive shelter dogs (*n* = 48) the lowest (median cpg 3.1 × 10^3^, IQR 1.1 × 10^3^–1.2 × 10^4^). The qPCR positive hunting dogs (*n* = 30) and household dogs (*n* = 47) showed intermediate shedding of cysts (median cpg 1.2 × 10^4^, IQR 6.4 × 10^3^–2.2 × 10^4^ and median cpg 5.4 × 10^3^, IQR 1.5 × 10^3^–1.3 × 10^4^, respectively).

## Discussion

Bayesian approaches have been used for comparison of commonly used diagnostic tests for *G. duodenalis* in dogs before, but the relatively recently developed qPCR has not yet been included [3, 28]. To calculate test performances in absence of both a gold standard and *a priori* information on infection status of the dogs, we performed a Bayesian latent class analysis, using the cross classified test results of CSF, DFA, IDEXX SNAP *Giardia*® and qPCR.

Three assumptions are made when using a Bayesian latent class model [23]. The first assumption is conditional independence given disease status. Since CSF and DFA both detect cysts, conditional independence seems unlikely, so conditional covariance for these two tests was included in the model. As the other two tests both use different approaches for identifying *G. duodenalis* presence, conditional independence can be assumed for these tests. Secondly, the model assumes constant sensitivity and specificity across the four populations. To evaluate this assumption, we ran the model, whilst excluding each dog population one by one. Specificity and sensitivity estimates were stable during this analysis, except for sensitivity of IDEXX SNAP *Giardia*®, which decreased to 60.0% when the clinical dogs were excluded (results not shown). This may suggest that the sensitivity of IDEXX SNAP *Giardia*® is higher in clinical dogs compared to the other populations. Indeed, we found the number of cysts per gram feces to be higher in clinical dogs, but why this did not affect the three other tests cannot be fully explained with our data. Finally, the model assumes differences in prevalence between populations, which is true for our data since the prevalence ranged from 7.9% in household dogs to 64.9% in hunting dogs.

Latent class models estimate the sensitivity and specificity based on test-outcome data from animals of unknown disease status. Thus, disease status is a latent variable which is defined by the combination of all tests used in the model. In our study, the four tests all rely on direct detection of *Giardia*, but do so in different ways. The qPCR is positive when *Giardia* DNA (SSU rDNA) is detected. IDEXX SNAP *Giardia*® is positive when soluble *Giardia* specific cyst wall antigen GSA-65 [29] is detected, and CSF and DFA when intact cysts are detected. In case of a positive test result, it does not automatically mean that the dog is infected with *G. duodenalis*. Because of the host specificity of *Giardia*, only ingestion of cysts of the appropriate *Giardia* species and assemblage can result in a patent gastro-intestinal infection. This means that ingestion of cysts of *G. duodenalis* assemblage A, B, C or D can lead to true infections in dogs. Ingestion of cysts of non-canid *Giardia* species, such as *G. muris*, *G. microti* or *G. psittaci*, or *G. duodenalis* assemblages E, F, G or H, will not lead to a patent infection in dogs and as a result, it is very unlikely that intact cysts will be present in the feces. Therefore, positive results with CSF and DFA generally represent true infection. However, it is possible to mistake other structures for *Giardia* cysts, which may lead to CSF and DFA false positive results. Given the lower specificity, this appears to occur more often with the DFA. Positive results of IDEXX SNAP *Giardia*® nearly always represent infection rather than mere passage, since this test had the highest specificity. The qPCR was originally developed for detection of *G. duodenalis* in human feces. The test characteristics of qPCR have been evaluated for human diagnostics, although not with latent class analysis [30]. For veterinary use, it is important to know which *Giardia* species and assemblage can be amplified. The dog, by its behavior, easily encounters all kinds of *Giardia* cysts*.* For coprophagy alone, it is estimated that at least half of the household dogs exhibit this behavior [20], let alone dogs that hunt, eat soil or drink from water puddles. So, dogs have a relatively high chance of picking up *Giardia* of any species and assemblage compared to environmental exposure of humans in developed countries. In our study, seventy out of the total of 573 samples (12.2%) tested positive with qPCR and negative in the three other tests. Based on the model estimates for the overall true prevalence (20.0%) and specificity of qPCR (85.6%), the expected total number of false positives in our samples was 66. Therefore, on average, 35% of all qPCR positive results (*n* = 189) might have been false positive. When assuming all false positives tested negative in the other three tests, 94% of the 70 uniquely qPCR positives may have tested false positive. To gain more insight whether the relatively low specificity of the qPCR was caused by false positive test results or represents false negative infection status and consequently an underestimation of the true prevalence, two additional experiments were conducted. Also, primers and probe of the qPCR were aligned with *Giardia* sequences available on GenBank. The results show that detection with the qPCR of non-canid assemblages E, F, G and H and of *G. psittaci* is possible. Literature shows that dogs are mostly infected with *G. duodenalis* C and D, followed by A and B [10, 31]. Indeed, the 12 samples of hunting and shelter dogs in additional Experiment 2 that could be sequenced, were all *G. duodenalis* assemblage D. It is therefore concluded that the low specificity of the qPCR in dogs is likely at least partially caused by the relatively low sensitivities of the other three tests used in the latent class analysis to identify the latent true disease status.

With the latent class analysis, qPCR had by far the highest sensitivity (97.0%), CSF the lowest (48.2%) and DFA (78.6%) and IDEXX SNAP *Giardia*® (71.9%) had intermediate sensitivity. The differences in median CPG at which the CSF, DFA and IDEXX SNAP *Giardia*® tested positive, were in accordance with these results. The results were also in accordance with the theoretical detection limits of qPCR (1.25 cysts per gram feces) and DFA (100 cysts per gram feces), but not with that of CSF (1–1.7 cysts per gram feces). The theoretical detection limit of CSF was vastly overestimated as our results show that CSF tested positive at a higher median CPG than DFA. It can therefore be assumed that the detection limits of CSF are in reality higher than the detection limit of the DFA. Detection limits for the IDEXX SNAP *Giardia*® test, which detects cyst wall proteins, could not be estimated in advance. From our results, it is likely that the theoretical detection limit of the IDEXX SNAP *Giardia*® is fairly similar to the detection limit of the DFA.

The qPCR detects DNA of cysts, intact or not, and trophozoites, which is, after DNA isolation, equally distributed throughout the tested sample. In the DNA isolation procedure, the DNA present in 0.2 gram of feces was eventually eluted in 100 μl. In combination with the DNA amplification steps this concentration step results in qPCR being the most sensitive diagnostic tests, although only a very small amount of the fecal sample is tested. As mentioned before, CSF and DFA depend on the detection of intact *Giardia* cysts. Although the cysts are notoriously robust, especially in water, in fecal samples they show a tendency to deteriorate relatively quickly [32]. Temperature and duration of storage of the fecal sample will therefore affect the actual detection limit and sensitivities of both CSF and DFA more than of enzyme immunochromatographic assay and qPCR. Also, *Giardia* cysts are sensitive to dehydration and might not be homogeneously distributed throughout the fecal sample. Since cyst shedding is not consistent [33], this further affects the sensitivity of tests relying on cyst detection. In this study, we processed fecal samples as soon as possible after arrival and to optimize storage conditions for DFA, SAF was added to the fecal sample in accordance to the manufacturer’s manual. Although performing the CSF and adding SAF (DFA) to the fecal sample was done as quickly as possible after collecting the sample, it could not be avoided that for some samples (especially the household and clinical dogs) this took more than a few days. By affecting the sensitivity of CSF and DFA, this could also have had a negative effect on the estimated prevalences of the household and clinical dogs. The sensitivity of qPCR could also have been affected, because fecal components are known to affect DNA in deteriorated cysts. However, considering the results of the model, when excluding each dog population one by one, the negative effects of the time it took before the samples were processed appeared not to be substantial.

The low sensitivity of the CSF microscopy on single day samples found in this study is in accordance with other studies [18, 28]. Performing the CSF on three-day samples, which is commonly advised for *G. duodenalis* diagnostics, would increase the sensitivity of CSF microscopy without a marked reduction in specificity using a parallel interpretation of the repeated testing [25]. Since microscopy is the most widely available test that provides information about a patent *G. duodenalis* infection in combination with additional information about the presence of other gastrointestinal parasites, this test remains very useful for both diagnostic and research purposes. It should be noted that the specificity of CSF microscopy for detection of *G. duodenalis* is high, provided it is performed by experienced examiners.

Commercially available point of care assays are fast, user-friendly and require little experience. They are therefore widely used in veterinary practice. With our latent class analysis, IDEXX SNAP *Giardia*® was shown to be highly specific (99.6%) but lacking sensitivity (71.9%). IDEXX SNAP *Giardia*® is registered for use in dogs and cats. This coproantigen test detects *G. duodenalis* assemblages C, D and F. According to literature, assemblages A and B are very likely detected as well [34, 35]. Compared to IDEXX SNAP *Giardia*®, DFA had a higher sensitivity (78.6%), which is in accordance with another Bayesian study [3].

Finally, for all tests, whether it concerns the highly sensitive qPCR or the least sensitive CSF, clinicians need to thoroughly consider the possible causes of a positive test together with the test characteristics to avoid over-diagnosing and over-treatment of *Giardia*. In case of a positive test result for *G. duodenalis* the dog has either giardiosis (infected and showing relevant symptoms such as diarrhea), giardiasis (infected but no symptoms) or is not truly infected. In the latter case, the test may be positive due to passage or contamination. In case of giardiosis, it can be expected that there generally will be high numbers of cysts in the feces. In such cases it is likely that the qPCR will become positive after fewer cycles (at lower Cp values). This is supported by the results showing that the higher the number of cysts detected with the CSF, the more unlikely it became that the other tests, including qPCR, were negative. Also, the samples uniquely positive for qPCR overall showed very low CPG levels.

## Conclusions

*Giardia duodenalis* is a prevalent gastrointestinal parasite in dogs, especially in hunting dogs. The qPCR is by far the most sensitive test for detection of *G. duodenalis* and therefore a useful tool for screening, although specificity of the qPCR was relatively low. Sensitivities of IDEXX SNAP *Giardia*® and DFA were lower than of qPCR, but because of their higher specificity and because of other test characteristics, such as information about true infection status and user friendliness, these tests are of value. CSF is the least sensitive test but remains valuable because it is the only test which provides information about other gastrointestinal parasites. It is also highly specific and provides information about true infection status.

## Additional files

Additional file 1: Raw data. (XLSX 39 kb)

Additional file 2: **Text**. Experiment 1: Confirming true positivity of samples only positive with the qPCR. **Figure S1.** SSU rDNA qPCR [5] performed as conventional PCR without probe. **Figure S2.** Serial dilution of template for SSU rDNA qPCR [5] performed as conventional PCR without probe. (DOCX 1781 kb)

Additional file 3: **Text**. Experiment 2: Confirming detection of *Giardia duodenalis* with the qPCR. (DOCX 36 kb)

Additional file 4: **Text**. Detection of *Giardia duodenalis* assemblages and other *Giardia* species with the qPCR. (DOCX 363 kb)
