# Supplementary material for: Comparing four diagnostic tests for Giardia duodenalis in dogs using latent class analysis
Source: Parasit Vectors. 2018 Jul 31;11:439. doi: 10.1186/s13071-018-3014-2 (PMC6069568; doi:10.1186/s13071-018-3014-2)
Supplement: Supplementary file 2 — Text. Experiment 1: Confirming true positivity of samples only positive with the qPCR. Figure S1. SSU rDNA qPCR [5] performed as conventional PCR without probe. Figure S2. Serial dilution of template for SSU rDNA qPCR [5] performed as conventional PCR without probe. (DOCX 1781 kb) [file 13071_2018_3014_MOESM2_ESM.docx]

**Additional file 2**

**Text**

*Experiment 1 – Confirming true positivity of samples only positive with the qPCR*

Seventy samples were positive in qPCR [5], but negative in the other three diagnostic tests (Table 1). To confirm that it was the 63 bp fragment of SSU rDNA fragment of *Giardia* that was detected in the qPCR, the same primers, reagents and conditions as used in the qPCR were also used in a conventional PCR. Only the probe was omitted from the conventional PCR. Subsequently, the PCR products were run on 2.5% agarose in TBE with 100 bp markers. All available samples positive in qPCR and negative in the other three diagnostic tests (n=63) were run in the conventional PCR together with 20 randomly selected samples that were negative for all diagnostic tests (Figure S1).

Two samples were omitted from calculations because there was less than 5µl eluate available for the conventional PCR. A 63 bp band was visible for 52/61 (85%) qPCR positive samples (median CPG 2.3x10^3^, range 1.0x10^3^-1.9x10^5^). For 9/61 (15%) qPCR positive samples (median CPG 1.3x10^3^, range 3.7x10^2^-2.7x10^5^) there was no such band. In none of the 20 qPCR negative samples a 63 bp band was observed after gel electrophoresis. In addition to the 63 bp band, there were many more bands visible. These bands were also visible in the 20 qPCR negative samples and, therefore, could not have caused the false positive qPCR results. Furthermore, similar results with the same PCR were obtained by Boadi et al. [27]. These additional bands were therefore most likely the result of omitting the probe in the conventional PCR.

**Figure S1**. SSU rDNA qPCR [5] performed as conventional PCR without probe.

Position of 63 bp product is indicated with an arrow.

CPG (+/-) = Cysts Per Gram and 63 bp product was detected (+) or not (-).

<5 µl = template less than 5 µl (result not used in calculations)

Positive control: purified cysts, equivalent to 10,000 CPG

A concentration dependent sensitivity was shown by making a serial dilution with two isolates (Figure S2). Dilutions equivalent to 960 CPG (0.8 cysts per PCR reaction) or higher were always positive for both isolates and showing increasing 63 bp band intensity with increasing CPG. The dilution equivalent to 192 CPG (0.16 cysts per PCR reaction) was positive for one isolate and negative for the other.

**Figure S2**. Serial dilution of template for SSU rDNA qPCR [5] performed as conventional PCR without probe.

Serial dilutions were made equivalent to 600,000 (1), 120,000 (2), 24,000 (3), 4800 (4), 960 (5) and 192 (6) Cysts per Gram (CPG). Position of 63 bp product is indicated with an arrow.

Positive control (P): purified cysts, equivalent to 10,000 CPG.
