# Supplementary material for: Comparing four diagnostic tests for Giardia duodenalis in dogs using latent class analysis
Source: Parasit Vectors. 2018 Jul 31;11:439. doi: 10.1186/s13071-018-3014-2 (PMC6069568; doi:10.1186/s13071-018-3014-2)
Supplement: Supplementary file 3 — Text. Experiment 2: Confirming detection of Giardia duodenalis with the qPCR. (DOCX 36 kb) [file 13071_2018_3014_MOESM3_ESM.docx]

**Additional file 3**

**Text**

*Experiment 2 – Confirming detection of* Giardia duodenalis *with the qPCR*

Dogs, especially hunting dogs, can take up cysts or trophozoites of *Giardia* species other than *G. duodenalis* by eating feces, prey animals or drinking surface water. Therefore, we wanted to confirm that a positive qPCR reaction was caused by *G. duodenalis* and not by other Giardia species. Consequently, we subjected all 35 samples from shelter and hunting dogs that tested positive with qPCR and negative with the other three diagnostic tests for *G. duodenalis* to a SSU rDNA PCR [22]. Twelve samples (34%) with a median of 7.2x10^3^ CPG (range 2.2x10^3^-1.1x10^6^) yielded a PCR product. They were all genotyped as *G. duodenalis* assemblage D. The remaining 23 samples with a median of 1.3x10^3^ CPG (range 1.4x10^2^-1.5x10^4^) tested negative in the SSU rDNA PCR. By making serial dilutions and spiking with positive samples (data not shown), it was shown that the SSU rDNA PCR had a sensitivity of 720 CPG. We concluded that in the samples tested positive with qPCR and negative with the other diagnostic tests no other Giardia than *G. duodenalis* could be found and that the SSU rDNA PCR [22] was unable to amplify the samples with lower CPG, due to low sensitivity.
