# Supplementary material for: Comparing four diagnostic tests for Giardia duodenalis in dogs using latent class analysis
Source: Parasit Vectors. 2018 Jul 31;11:439. doi: 10.1186/s13071-018-3014-2 (PMC6069568; doi:10.1186/s13071-018-3014-2)
Supplement: Supplementary file 4 — Text. Detection of Giardia duodenalis assemblages and other Giardia species with the qPCR. (DOCX 363 kb) [file 13071_2018_3014_MOESM4_ESM.docx]

**Additional file 4**

**Text**

*Detection of* Giardia duodenalis *assemblages and other* Giardia *species by the qPCR*

The 63 bp products of the PCR were too short to get good sequence results. To check for possible matches, we aligned the forward primer (5′-GACGGCTCAGGACAACGGTT-3′), the reverse complement sequence of the reverse primer (5′-TTGCCAGCGGTGTCCG-3′) and probe (FAM-5′-CCCGCGGCGGTCCCTGCTAG-3′-TAMRA) of the qPCR [5] with in GenBank available *Giardia* sequences.

Sequences of in GenBank available *Giardia* species, aligned to the forward and reverse primers and probe of the qPCR.


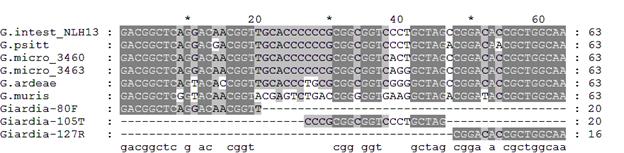


Giardia-80F = forward primer; Giardia-105T = probe; Giardia-127R = reverse primer

GenBank accession numbers: G. intest_NLH13 AY826201; G. psitt AF473853; G. micro_3460 AY228333; G. micro_3463 AY228332; G. ardeae M73684; G. muris M73682

Sequences of *G. duodenalis* assemblages A to G and subassemblages AI, AII, AIII, BIII and BIV, aligned to the forward and reverse primers of the qPCR.

Note that the probe (bp 82-101) is not aligned.

GenBank accession numbers: AI_sprong M54878; AII_sprong AF199446; AIII_sprong DQ100287; BIII_sprong AF199447; BIV_sprong AF113898; C_sprong AF199449; D_sprong AF199443; E_sprong AF199448; F_sprong AF199444; G_sprong AF199450

The results suggest that *G. duodenalis* assemblages A to G and possibly also *G. psittaci* (birds) can be detected with the SSU rDNA qPCR. The detection of other *Giardia* species, such as *G. ardeae, G. microti* and *G. muris,* is less likely. The recently recognized *G. duodenalis* assemblage H (seals, gulls) will very likely also be amplified with the qPCR since it is closely related to the other assemblages [26].
